# The visual effect of wind turbines on property values is small and diminishing in space and time

**DOI:** 10.1073/pnas.2309372121

**Published:** 2024-03-18

**Authors:** Wei Guo, Leonie Wenz, Maximilian Auffhammer

**Affiliations:** ^a^CMCC Foundation - Euro-Mediterranean Center on Climate Change, Leece 73100, Italy; ^b^RFF-CMCC European Institute on Economics and the Environment, Centro Euro-Mediterraneo sui Cambiamenti Climatici, Milan 20144, Italy; ^c^Department of Complexity Science, Potsdam Institute for Climate Impact Research, Potsdam 14412, Germany; ^d^Mercator Research Institute on Global Commons and Climate Change, Berlin 10829, Germany; ^e^Department of Agricultural and Resource Economics, University of California, Berkeley, CA 94720; ^f^National Bureau of Economic Research, Cambridge, MA 02138-5398

**Keywords:** renewable energy, economic valuation, statistical analysis, climate change mitigation

## Abstract

Renewable power generation is the key to decarbonizing the electricity system. Wind power is the fastest-growing renewable source of electricity in the United States. However, expanding wind capacity often faces local opposition, partly due to a perceived visual disamenity from large wind turbines. Here, we provide a US-wide assessment of the externality costs of wind power generation through the visibility impact on property values. To this end, we create a database on wind turbine visibility, combining information on the site and height of each utility-scale turbine having fed power into the U.S. grid, with a high-resolution elevation map to account for the underlying topography of the landscape. Building on hedonic valuation theory, we statistically estimate the impact of wind turbine visibility on home values, informed by data from the majority of home sales in the United States since 1997. We find that on average, wind turbine visibility negatively affects home values in an economically and statistically significant way in close proximity (<5 miles/8 km). However, the effect diminishes over time and in distance and is indistinguishable from zero for larger distances and toward the end of our sample.

## Significance

A substantial expansion of renewable energy generation is necessary for decarbonizing the U.S. economy. Wind power is the fastest-growing renewable source of electricity in the United States. It has been argued that wind turbines are a visual disamenity. We statistically estimate the impact of having at least one wind turbine within sight on home values, using data from more than 300 million home sales and 60,000 wind turbines in the United States from 1997 to 2020. We find robust evidence of a 1% drop of home values within a wind turbine’s viewshed. The effect is larger for homes closer to more wind turbines, but is no longer detectable by the end of the 20-y period covered by our data.

Investment in renewable power generation capacity has gained significant momentum in the United States and globally in recent years (1). This is driven by massive drops in the cost of wind and solar and by concerns over the negative local and global externalities stemming from a fossil fuel–based energy system. While renewable technologies address the issue of pollution externalities, their rollout poses different challenges (e.g., intermittency) (2, 3). Wind power is the fastest-growing source of renewable electricity in the United States. In 2020, wind power accounted for more than 7% of total electricity generation, and it is projected to continue to grow in the coming years (4).

Wind turbines, in particular, have also been a source of controversy as they may create low-frequency noise, cast shadows, create flickering, and visually degrade the landscape (5–8). Understanding the visual disamenity value of wind turbines is becoming increasingly policy relevant as already large wind turbines are growing in height and are often located on high-elevation areas with extensive visibility (9, 10). They are widely perceived as unattractive and disruptive to the landscape, with some polls suggesting that 8 to 25% of respondents strongly dislike seeing wind turbines (11, 12). Homeowners and developers may be negatively affected by the proximity of wind turbines through depressed home values (13–15). These “NIMBY” (Not In My BackYard) concerns, which in many places have manifested in vocal local public opposition to new projects, can have an impact on the siting decision of wind power infrastructure (16–18).

**Fig. 1.**
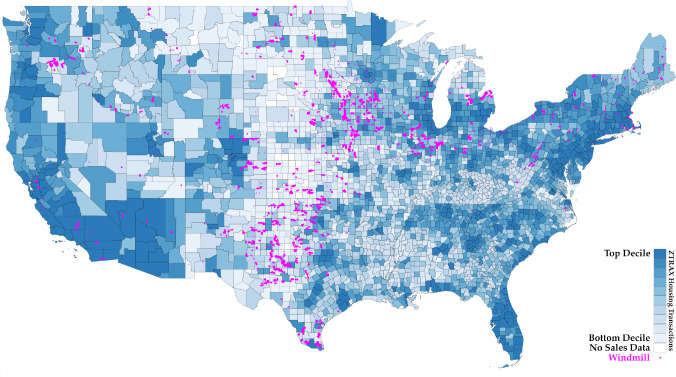
Map of property transactions and wind turbine locations. Blue shading indicates the number of property transactions between 1990 and 2020 by decile, aggregated to the county level. Areas without sales data are white. Magenta dots show the locations of wind turbines installed in that period.

This paper presents a comprehensive national-level analysis to causally estimate the visual externality costs of wind power generating capacity in the United States. We utilize the universe of re-geocoded home transactions listed in the ZTRAX database for the years 1997 to 2020 and match these to the installation of wind turbines nearby (Fig. 1).

We rely on the broadly applied theory of hedonic valuation to reveal local residents’ preferences for views of wind turbines (19, 20). Previous studies have either focused on wind facilities outside of cities in Europe or on selected areas across the United States, making their results difficult to generalize to the entire USA (18, 21–27). Further, unlike previous studies, we do not only consider mere proximity of a wind turbine to a home, but compute whether a wind turbine can actually be seen from each home (visibility). To this end, we combine digital elevation models of the landscape with the location and height information of turbines, utilizing advanced geospatial tools from geomorphometry and computer science (28–30). We can thus create a geospatial database on wind turbine visibility, comprising a high-resolution viewshed for every single wind facility in the USA (see *Data & Methods* for detail). This database allows us to characterize whether and when a location can actually see a wind turbine or whether it is hidden from view by the natural landscape.

To investigate the causal effect of wind turbines on housing prices, we employ a spatial difference-in-difference (DiD) design that takes advantage of both temporal variation in turbine installations and spatial variation in visibility induced by the underlying topography of the landscape. Our analysis estimates the average change in housing prices for homes with a wind turbine in their viewshed when it becomes operational, relative to the average change in housing prices for homes not visible to the same facility, within a 10-km (6.2-mile) range from the wind facility. The high-resolution housing transaction data, which include precise property locations, allow us to relax the statistical identification assumption, as the exact location and installation of wind turbines is assumed to be exogenous to the evolution of nearby housing markets. This is because the visibility of wind turbines is primarily determined by the underlying landscape topology, which is exogenous to changes in property values over time. Our examination of the parallel property value trends assumption pre-installation supports this statement. We also control for other confounding variables, such as location, general economic trends, and housing quality (see *Data & Methods* for detail).

## Results

### Property Value Impacts.

We find that having at least one wind turbine in a home’s viewshed (10 km radius) reduces the sales price of such a property on average by 1.12%, which is statistically different from zero Table 1, column (1). To put this in perspective, this amounts to a US $24.5 billion reduction in the property value for all houses affected by the visibility disamenity effect nationwide, which is small when compared to the total value of US homes (≈ US$45 trillion).

**Table 1.** Baseline regression results of wind turbine visibility on property value

Log(Property Value)	(1)	(2)	(3)	(4)
Treated x Post	−0.0112** (0.0043)	−0.0090** (0.0042)		−0.0103** (0.00415)
x # Turbines (x10)		−0.0021*** (0.0005)		
x # Turbines (<20)			−0.0102** (0.0042)	
x # Turbines (>20)			−0.0248** (0.0096)	
x Installation Year				0.00169** (0.000683)
Post-treatment	0.0090 (0.0059)	0.0102* (0.0058)	0.0098* (0.0058)	0.0104* (0.00563)
Treated	-0.0101** (0.0044)	−0.0101** (0.0044)	−0.0101** (0.0044)	−0.0101** (0.0044)
*N*	5,705,597	5,705,597	5,705,597	5,492,914
Adj. R^2^	0.516	0.516	0.516	0.515
Property Char.	X	X	X	X
Census tract x year	X	X	X	X
County x month	X	X	X	X

Column (1) shows the effect of wind turbine visibility on average property values within 10 km. The control group includes properties that are within 10 km but cannot see any wind turbine. Coefficients on property characteristics are reported in column (1) of *SI Appendix*, Table S1. Column (2) differentiates between the effect of the first wind turbine in view and that of every additional 10 wind turbines (second row). Column (3) distinguishes between the effect of less than or more than 20 wind turbines, respectively. Column (4) incorporates the trend interaction of the DiD indicators with the demeaned installation year of wind turbine (mean is 2011).

Additionally, we estimate that prior to the installation of a wind facility, there is a significant gap of 1.01% in the average property value between those areas that will later have a wind turbine in their viewshed (treated areas) and those that will not (control areas). This gap cannot be explained by differences in observed property characteristics or disparities in neighborhood factors and housing market changes. Our best explanation for this gap is that wind turbines are more likely to be sited in areas where their visual disamenity affects communities with lower housing values. Since the visibility of wind turbines is primarily determined by the nearby geographic landscape, this cross-sectional gap reflects only subtle decisions on wind turbine location within a small area rather than across entire neighborhoods.

The finding regarding the disamenity effect remains robust across multiple ways of specifying the regression model. These include limiting the sample to only properties that have experienced repeated sales over the research period, excluding nondisclosure states on property transactions, incorporating interaction terms between property characteristics and yearly indicators, as well as interacting treatment with state indicators. These checks are reported in *SI Appendix*, Table S1.

We investigate whether the visual impact of wind turbines varies with the intensity of visibility using two measures: The number of wind turbines in view and the intensity classified by whether there are more than 20 turbines in sight. We find that the capitalization of the visual disamenity increases with the treatment intensity, with every additional 10 wind turbines in view reducing the property value by an additional 0.2% [Table 1, column (2)]. Furthermore, wind farms with more than 20 turbines reduce the property value in visible areas by an average of 2.48%, whereas those with less than 20 turbines have a reduction effect of only 1.02% on visible areas [Table 1, column (3)]. These findings suggest that the density of wind turbines in view plays a role in driving the magnitude of the visual disamenity valuation.

**Fig. 2.**
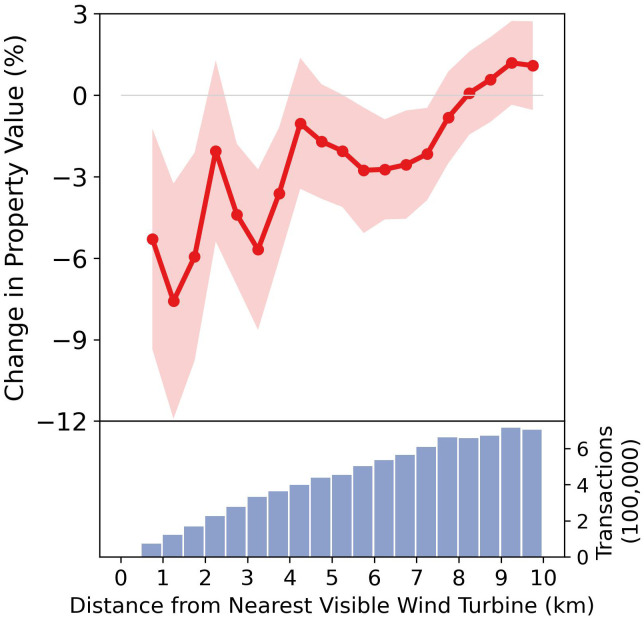
Effect of wind turbine visibility on property value by distance. The effect of wind turbine visibility on property values for different distance bins. Each distance bin represents a 500-m range, determined by the distance from the nearest visible wind turbine. Red dots indicate estimated coefficients, as obtained by interacting the coefficient of interest from the difference-in-difference model (interaction between post-treatment status and treatment assignment) with distance bin indicators. Shaded areas represent 95% CIs constructed using two-way clustered SEs at the census tract and year level. Bars in the *Lower* panel show the number of transactions within each distance bin, corresponding to the right y-axis.

The impact of visual disamenity created by wind turbines may also vary depending on the distance from the nearest visible turbine. To test how the effect varies by distance, we re-run the baseline specification with the indicators of interest interacted with 500-m (∼ 0.3-mile) distance bin indicators for the proximity of the closest wind turbine. The effect of wind turbine visibility decreases as distance increases (Fig. 2). The effect is largest in immediate proximity of wind turbines—with the visual disamenity reducing property values by up to 8% within a neighborhood range of 1.5 km (∼ 0.9 miles). Even though this number is economically large, there are two noteworthy caveats. First, the CI is sizable including reductions in property value between 3 and 13%. Second, the number of properties within this distance bin is small. Nationally, there are fewer than 250,000 transactions within 1.5 km of the nearest wind turbine, as opposed to approximately 8.5 million transactions within 10 km. The effect for the full sample is statistically indistinguishable from zero 8 km (5 miles) away from the nearest wind turbine. To put this in perspective, if one stretched out an average-sized arm and held up an aspirin tablet, this would equate the perceived size of an average wind turbine five miles away. Were the same wind turbine one mile away, it would appear to be roughly the size of a golf ball.

To further test the robustness of our results to model setting, we modify the baseline DiD specification in various ways, using single, triple, and quadruple difference frameworks, respectively (*SI Appendix*, Table S2). First, we conduct a spatial difference by visibility and by proximity (within 10 km) only, without using the installation timing (columns 1 and 2). Second, we expand the sample to 50-km distance and identify the treatment coefficient by a 10-km proximity interaction (column 4). Finally, within this last framework, we also allow for effects of proximity to interact with installation timing (column 5). The results are almost identical to the effect of visibility to wind turbines on property value found in the baseline model (column 3). Moreover, we find that properties within 10 km from wind turbines are 1.16 to 1.7% lower in sales price than those 10 to 50 km away. These gaps are not driven by differences in the housing characteristics of properties located in different communities. Therefore, the cross-sectional difference in property value between visible and nonvisible areas as well as between proximate and distant areas indicates a potential selection effect that is consistent with the siting of wind turbines in places with lower property values.

As our visibility metric only considers features of the landscape and not buildings, we restrict the sample to areas with low building heights (*SI Appendix*, Fig S1). The results from this analysis are given in *SI Appendix*, Table S3. The results are almost identical to those in our main specification, with a point estimate of −1.2% for urban areas with low building heights.

**Fig. 3.**
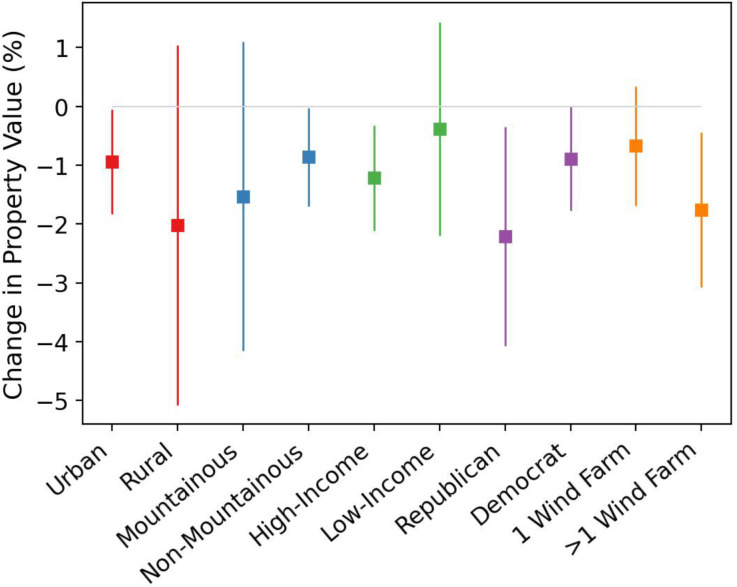
Visual disamenity effect of wind turbine visibility by different county characteristics. Squares are the point estimates of the effect of wind turbine visibility on property values when partitioning the data based on different county characteristics. These estimates stem from a regression model, which adjusts the baseline model in Column (1) of Table 1, incorporating an interaction between the treated-post-treatment indicator with the categorical indicator of interest. Results for urban and rural counties, respectively, are shown in red. Blue indicates a segmentation into mountainous and nonmountainous groups, having compared the county’s average elevation with 1,000 m. Green gives results having split counties by whether the median household income is above the nation’s average level, using the 2015 American Community Survey. Purple divides counties between right-wing and left-wing using the presidential election results from the 2016 election. Orange breaks counties down by whether there is only one wind farm or multiple farms. The 95% CIs of the estimates are shown as bars, having clustered SEs at the census tract and year level. The zero effect line is illustrated by a gray line for reference.

We further investigate whether the visual disamenity effect varies across various dimensions, as shown in Fig. 3. We find that the negative impact of wind turbines on property values is primarily observed among urban properties, with negative but noisy effects in rural areas. Our analysis based on geographical altitude suggests that the negative impact of wind turbine visibility is particularly pronounced in nonmountainous regions. We also observe a strong correlation between local political leanings and disamenity effects, with right-leaning communities experiencing a significantly greater impact compared to left-leaning areas. Last, the visual disamenity is more accentuated in high-income locales as opposed to low-income areas.

**Fig. 4.**
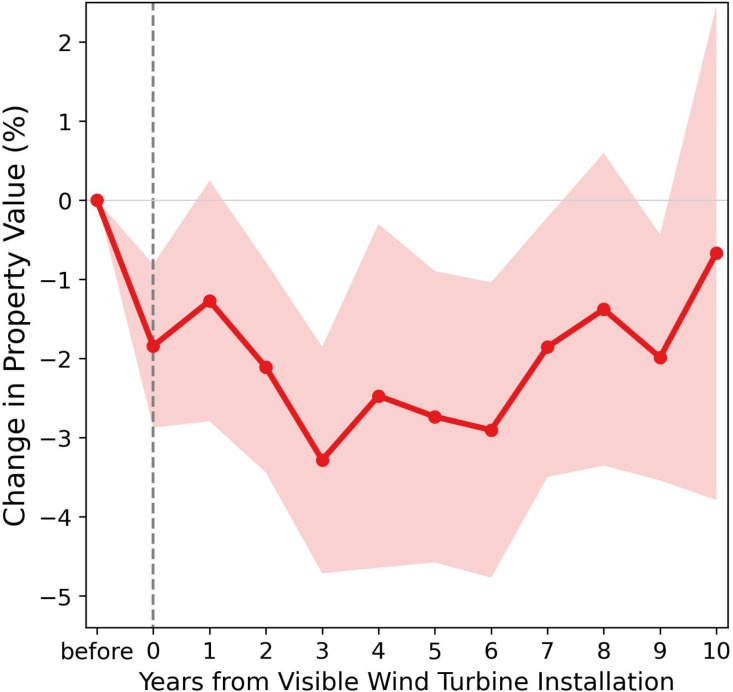
The effect of wind turbine visibility on property values for different year bins, relative to the year when the wind turbine was installed. Each bin represents 1 y, determined by the year of property transaction relative to the year of the first installation of a wind turbine in visibility. Black dots are the estimated coefficients, as obtained by interacting the coefficient of interest from the difference-in-difference model (interaction between post-treatment status and treatment assignment) with the year indicators. The coefficients for pre-installation periods are normalized to zero. Shaded areas represent 95% CIs constructed using two-way clustered standard errors at the census tract and year level.

The impact of wind turbine visibility may not manifest immediately, as it could require time for people to perceive and adapt to the visual disamenity (14). We explore the durability and dynamics of this effect in three ways. First, we investigate the impact trajectory following a wind turbine installation (Fig. 4). We find that the disamenity impact emerges instantly upon the installation, leading to a decline of more than 3% in nearby property values over the following 3 y. The effect diminishes and eventually disappears within the next 7 y. This is consistent with the idea that people initially dislike wind turbine installations but gradually become accustomed to them over time. Consistent with that, we find in a second analysis that wind turbines installed in earlier periods have a significantly more pronounced visual disamenity effect than more recent installations (column 4 of Table 1). We arrive at this finding by incorporating interaction terms between a trend of installation year and our DiD indicators (*Methods*). This is a significant finding, suggesting that the average effect we estimate above is larger than the effect one would expect for recent and future installations. Specifically, while an average wind turbine (installed in 2011) has a negative effect on nearby property values, the effect becomes indistinguishable from zero for turbines installed after 2017. Our third analysis differentiates the disamenity effect between initial and subsequent wind turbine installations. We segment the data based on years and counties that have seen a county’s first wind farm installation and conduct the baseline regression separately for the two groups (orange squares in Fig. 3). We find that the introduction of the first wind farm results in an insignificant, minor effect, whereas subsequent installations lead to a more substantial, noticeable decrease in property values.

## Discussion

This paper provides a national-level plausibly causal evaluation of the externality costs of wind power generation through the visibility impact on property values in the United States. We take advantage of the densely populated geographic setting across the nation, with rich geological features such as undulating terrain and prominent elevations on the surface, and numerous wind farms developed within sight of residential properties. We use advanced geospatial tools from geomorphometry and computer science to overcome computational difficulties and construct a comprehensive database on wind turbine visibility throughout the nation. Our analysis relies on the universe of housing transactions in the ZTRAX database spanning a 20-y period across the country and employs a spatial difference-in-difference design based on a quasi-experimental setting that compares the effect of wind power installation on property values in areas visible to the turbines with the value change of properties within the same area—but not visible to the same facility.

The findings indicate that wind turbines have a negative effect on property value in locations where they are visible. On average, across the whole sample, house prices decrease by up to 8% after the construction of a wind turbine within viewshed and close neighborhood range from the property, with the effect decaying as the distance increases. The average effect falls to a 1% reduction for houses within 10 km of visible wind turbines. It also diminishes over time—both in terms of more recent installations having a smaller disamenity effect and in the sense that the reduction in value a property experiences peaks 3 y after the installation and then becomes smaller the more years pass. These findings are consistent with a cognitive model where people get used to new structures in their environment over time.

The reduction in property values resulting from wind turbine installations raises questions about how this might affect siting decisions for future wind farms. This paper highlights the externality of wind power developments as they are capitalized in the housing markets. These estimates could also serve as a future basis for calculating compensation to local homeowners for placing a new wind turbine within their viewshed.

### Data & Methods.

The analysis primarily utilizes data from three sources: the wind turbine installation panel, the real estate transaction records, and the digital elevation models.

#### Wind turbine operation.

We obtain the full sample of wind turbine installations from the United States Wind Turbine Database (2022 Version) of the United States Geological Survey (USGS), which has collected and compiled comprehensive records of wind turbines from various public and private sources on a quarterly basis. The data consist of all land-based and offshore utility-scale turbines that have ever generated and fed power into the grid to supply utilities with energy, including both newly installed as well as dismantled ones across the nation. For each facility, the data provide geo-referenced information of longitude and latitude, dates of announcement, construction, and operation, along with technical specifications on turbine make and model such as nameplate capacity, hub height, rotor diameter, and facility size.

We limit the sample to wind turbines that have started installation or have been in operation anytime since 1997. This includes 68,649 facilities in the continental United States, with their summary statistics presented in *SI Appendix*, Table S4.

#### Housing transactions.

Data on the universe of property transactions are obtained from the ZTRAX database (2021 version). The data are created by combining transaction observations from multiple sources, including records from the buyer’s, seller’s, and county assessor’s points of view, along with records from county assessments on an annual basis. This results in a rich dataset that allows us to observe the date and sale price of each transaction, as well as key characteristics of the transacted property, such as the property type, year of construction and renovation, building area, number of bedrooms and bathrooms, and other amenity features included in assessments. Each property or parcel point is geographically identified by its street address, and we conduct geocoding using the USA Local Composite locator of ArcGis to obtain the exact geo-referenced location. The data cover records of housing transactions from 1997 to 2020.

To conduct hedonic valuation, we limit our analysis to residential properties within the continental US, and exclude non-arm’s-length transactions (below $10,000) or outlier properties (above $4,000,000), which account for 3.1% of the total data. We also exclude transactions that occurred on the same parcel within 3 mo of the previous sale to avoid duplicate observations. To examine the impact of visibility disamenity on property values, we further limit our sample to properties within 10 km of wind turbines, as discussed below. The final data comprise 180,682,544 transactions, and their summary statistics are presented in *SI Appendix*, Table S5. The shares of treated properties and treated properties post treatment by state are presented in *SI Appendix*, Fig. S4.

#### Data for heterogeneity analysis.

We utilized data from the 2015 American Community Survey to obtain median household incomes at the county level. Counties were classified into high-income and low-income groups by comparing their median income with the national average.

Political leanings of counties were determined using the 2016 presidential election outcomes sourced from the MIT Election Data and Science Lab (31). Counties were categorized as right-leaning or left-leaning based on whether the majority of their voters chose the Republican or Democratic Parties, respectively, in the 2016 presidential election.

We acquired average elevation data at the county level through calculations based on Environmental Systems Research Institute data from the USGS. Counties were classified into mountainous and nonmountainous categories based on whether their average elevation exceeded 1,000 m. For context, the least elevated mountainous U.S. state, Montana, has an average elevation of 1,036 m. This categorization yielded 403 mountainous counties, representing 12.8% of all U.S. counties.

#### Digital elevation models.

Digital elevation models (DEMs) provide crucial information on the ground topography of the study area. The DEMs we utilize are based on the Shuttle Radar Topographic Mission produced by NASA, which employs remote sensing technology to gather laser light measurements of the earth’s surface. The resulting height (x), width (y), and depth (z) measurements are then used to create a comprehensive and accurate map of elevation for the entire globe. In particular, we use the most recent version of the DEMs data (2018), which are available at a resolution of 90 m for the entire continental US (32). The high level of accuracy and resolution of the DEMs is essential to our analysis, as it allows us to capture subtle variations in elevation and terrain that could have a significant impact on the sight of view.

#### Viewshed analysis.

One of the key contributions of our analysis is the creation of a comprehensive database that measures the visibility of wind turbines across the United States. This is achieved by generating and aggregating viewsheds from the location point of each wind turbine. Viewshed is a term used in geography and cartography that refers to the area visible from a specific observation point or vantage point, based on the topography of the surrounding terrain and any obstructions that may block the view. Unlike typical viewshed analyses that calculate the viewshed to each property, we compute the viewshed from the site of wind turbines thanks to the duality of vision, which requires less computational effort since the number of wind turbines is much smaller than the number of housing properties. This approach greatly increases computational efficiency.

Our analysis of viewshed generation involves combining the site and height data of each turbine with information on the underlying topography of the landscape and the curvature of the earth. By utilizing the viewshed module in GRASS GIS, we are able to differentiate neighboring residential properties based on their ability to view the facility. The module relies on the line of sight and its geographical intersection with the terrain, which offers considerable advantages in terms of accuracy, reliability, and efficiency.

The visual significance of an object decreases as its distance from the observer increases, and increases as the observer’s location elevates or as their height increases. To account for air quality conditions across the nation, we assume a maximum visible range of 10 km. Given that the horizontal distance of observation and the hub height of wind turbines are significantly greater than the height of a representative person, the observer’s height is unlikely to have a significant effect on the visibility analysis. Therefore, we assume a representative observer’s height of 1.75 m.

**Fig. 5.**
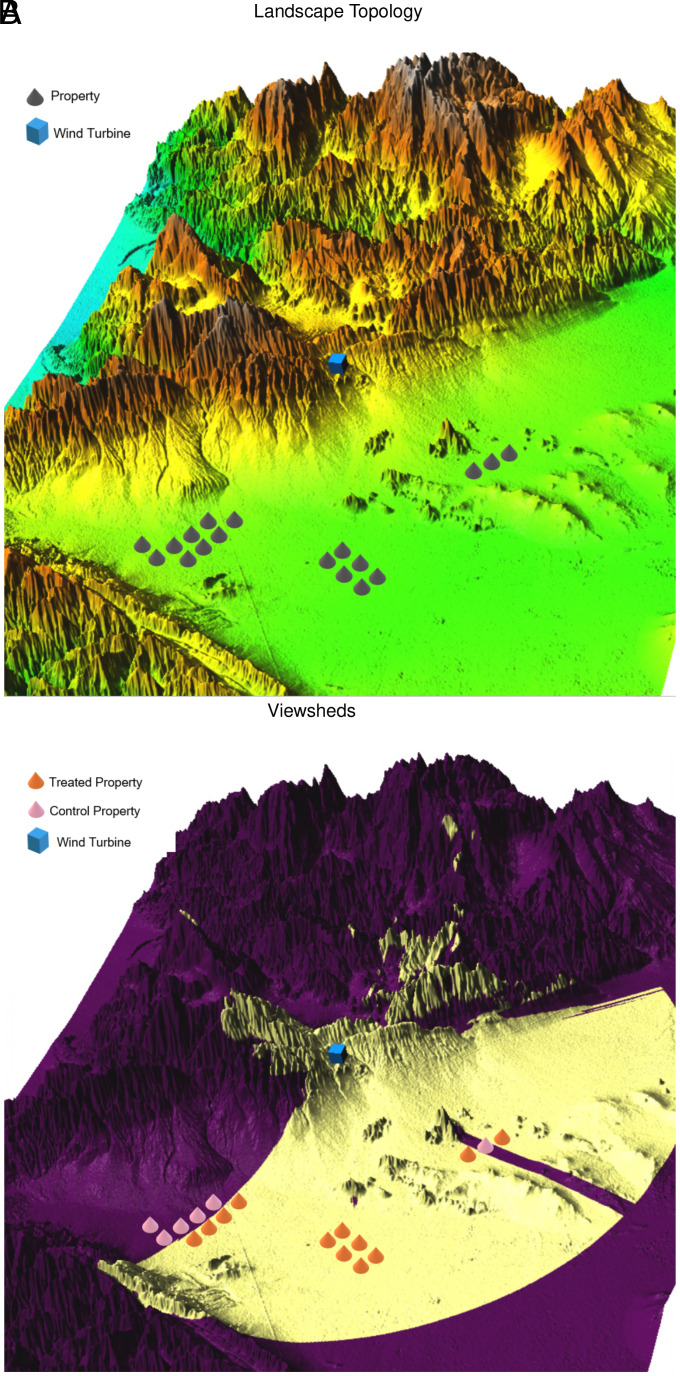
Surface and viewshed of patterson pass wind in Altamont of California. Panels (*A* and *B*) depict the landscape topology and viewshed, respectively, of a wind turbine located in the Patterson Pass Wind facility in Altamont, California. It has a hub height of 24 m, and rotor diameter of 16 m. The blue point located at the *Center* of both figures represents the wind turbine, and the gray cones represent housing properties. In Panel (*B*), light-colored areas indicate locations from which the turbine is visible, while dark-colored areas indicate areas where the turbine is not visible. Accordingly, orange cones are properties in the treatment group whereas pink cones are housing properties in the control group.

To visually illustrate the visibility analysis, we present an example of viewshed generation for a wind turbine in Fig. 5. This is a wind facility of the Patterson Pass Wind in Altamont, California, which became operational and began providing power in 1985, has the turbine capacity of 65 kw, the hub height of 24 m, and rotor diameter of 16 m. This is a fairly typical wind farm development in the full sample. Panel *A* illustrates the topographic features of the neighboring surface to the facility, which is represented by the blue point of center. Located in an approximately 50,000-acre area that extends across the northeastern hills of Alameda County and into a small portion of Contra Costa County to the north, the facility finds the visibility sight to itself largely void by the mountain ridges at high elevations in the north and remains visible from the south side only. This can be seen in Panel *B*, where the dark shaded areas represent places from which the view to the facility is obscured by geographical elevations, and the light yellow shading indicates lands where the hub of turbine blades is visible. Empirical results presented above rely on comparisons of outcomes occurring with the start of wind farm operation in the areas where the turbines are visible, and those occurring where they are nonvisible.

As of the end of 2020, wind power facilities have led to visual disamenity across more than a quarter of the continental US (*SI Appendix*, Fig. S2), resulting in exposure for approximately 37.2 million homes, which account for 30.6% of all households in the nation. A significant proportion of the affected populations are “treated” by multiple wind power facilities, with more than 75% of the affected lands exposed to the visibility of more than 10 wind turbines.

#### Statistics: Property value model.

We start by utilizing a standard difference-in-differences (DiD) framework to compare the effects of wind farm development on the property value of homes in visible areas after the wind turbines become operational, relative to places within the same area (i.e., within a radius of 10 km of the wind turbine in question) where the turbines are not visible. Our examination of pre-installation property value trends supports the assumption for paralleling pre-treatment trends, as shown in *SI Appendix*, Fig. S1. The specification is as follows:

[1] $$\begin{array}{cc} \log(P_{\mathrm{it}}) & =\beta_{1}\;{\text{Treat}}_{i}\times \;{\text{Post}}_{\mathrm{it}}+\beta_{2}\;{\text{Treat}}_{i}+\beta_{3}\;{\text{Post}}_{\mathrm{it}}\\ & \;\;+\beta_{4}X_{\mathrm{it}}+\alpha_{\mathrm{ny}}+\alpha_{\mathrm{cm}}+\epsilon_{\mathrm{it}}. \end{array}$$

Here, each observation corresponds to a transaction for property i that occurred on date t, with the outcome variable being the log of sales price $P_{\mathrm{it}}$. $\;{\text{Treat}}_{i}$ is an indicator that denotes whether a property was assigned to the “treated” group, which refers to whether the property is located in areas with any wind turbine in view either currently or in the future. Note that the division between treatment and control group depends only on the location of property i, rather than on the transaction date or wind turbine installation status. $\;{\text{Post}}_{\mathrm{it}}$ is the indicator that denotes whether a property was transacted after the wind turbine in view became operational. Thus, the coefficient $\beta_{1}$ for the interaction term between $\;{\text{Treat}}_{i}$ and $\;{\text{Post}}_{\mathrm{it}}$ captures the effect of wind turbine installation on the value of visible properties. To account for potential changes in building characteristics that could affect property values, we include several property characteristics $X_{\mathrm{it}}$ that could vary over time, including the most recent year the property was built or renovated, the number of bedrooms and bathrooms, and the lot size in acres.

Crucially, there might exist time-varying location-specific factors that correlate with the visual disamenity created by wind farms. For instance, the spatial distribution of pre-existing wind turbines may influence the siting decisions for future wind turbines, potentially due to the participation of local communities in policy making. This correlation might also exist when wind farms are not randomly assigned across space, or if areas close to wind farms where turbines are visible may not be comparable to those further away in terms of other amenities affecting housing prices. To address this, we assume that each community has its own time trend, fully capturing any community-specific factors that impacted the siting of wind turbines. We include these trends in the analysis by incorporating fixed effects on census tract by year level, denoted as $\alpha_{\mathrm{ny}}$ for census tract n and year y. Moreover, to control for seasonal trends in housing markets that might be specific to each county, we include fixed effects on the county by month level, denoted as $\alpha_{\mathrm{cm}}$ for county c and sales month m. This way, we can ensure the DiD estimator of interest, $\beta_{1}$, is not contaminated by correlation with the time effects driven by the endogenous selection of wind farm siting and the general trends in property value over time. It is worth noting that while we acknowledge that the siting decision of windmills might be correlated with the post-treatment trends in housing markets, our control for the census-tract-by-year and county-by-month trends will ensure the DiD coefficients to capture only nuanced location variations within a community, rather than pronounced variations across neighborhoods.

## Supplementary Material

Appendix 01 (PDF)

## Data Availability

Windmill data have been deposited in Github (33). Some study data available (Zillow’s Transaction and Assessment Database (ZTRAX) data: ZTRAX data are offered by Zillow’s research team through their website https://www.zillow.com/research/ztrax/ (34). To access ZTRAX data, the user will need to first review and agree to the ZTRAX Data License Agreement, then complete the registration online. Once logged in, the user can request access to specific ZTRAX datasets. For acquisition, the user will need to be prepared to provide details on the intended use of the data. To replicate of our study, we recommend applying for comprehensive access to ZTRAX’s housing transaction data, covering all U.S. states from 1995 to the most recent available data. If that fails, alternative housing data are provided by Corelogic, which can be purchased. Windmill data: Windmill data can be accessed from the United States Wind Turbine Database produced by United States Geological Survey (USGS), available at https://doi.org/10.5066/F7TX3DN0 (35). We have consolidated key data and saved it as windmill.dta in the replicate kit. Digital Elevation Models Digital Elevation Models are produced by NASA’s Shuttle Radar Topographic Mission and available at https://srtm.csi.cgiar.org/ (36). These are crucial for viewshed computations in the replication. Due to size constraints, the original Digital Elevation Models (DEMs) are not included as part of our replication kit. The user will need to download and extract the data to /data/DEMs/ under the replication directory. Other Data: Data for heterogeneity analysis are drawn from multiple sources. The county-level median household income records come from the 2015 American Community Survey. Presidential election data is sourced from the Massachusetts Institute of Technology (MIT) Election Data and Science Lab (37). Additionally, the county-level average elevation data is derived from the Environmental Systems Research Institute. All these data are either acquired through public access in our code or included in the replication kit.

## References

[1] I. E. Agency, “World energy investment 2022” (Tech. Rep., National Bureau of Economic Research, 2022).

[2] H. Westlund, M. Wilhelmsson, The socio-economic cost of wind turbines: A Swedish case study. Sustainability **13**, 6892 (2021).

[3] G. Gowrisankaran, S. S. Reynolds, M. Samano, Intermittency and the value of renewable energy. J. Polit. Econ. **124**, 1187–1234 (2016).

[4] X. Costoya, M. DeCastro, D. Carvalho, M. Gómez-Gesteira, On the suitability of offshore wind energy resource in the United States of America for the 21st century. Appl. Energy **262**, 114537 (2020).

[5] J. H. Schmidt, M. Klokker, Health effects related to wind turbine noise exposure: A systematic review. PloS One **9**, e114183 (2014). 25474326 10.1371/journal.pone.0114183 PMC4256253

[6] M. I. Dröes, H. R. Koster, Wind turbines, solar farms, and house prices. Energy Policy **155**, 112327 (2021).

[7] R. Saidur, N. A. Rahim, M. R. Islam, K. H. Solangi, Environmental impact of wind energy. Renew. Sustain. Energy Rev. **15**, 2423–2430 (2011).

[8] C. Krekel, A. Zerrahn, Does the presence of wind turbines have negative externalities for people in their surroundings? Evidence from well-being data. *J. Environ. Econ. Manag.* **82**, 221–238 (2017).

[9] E. J. Lantz *et al*., “Increasing wind turbine tower heights: Opportunities and challenges” (Tech. Rep., 2019).

[10] H. Alphan, Modelling potential visibility of wind turbines: A geospatial approach for planning and impact mitigation. Renew. Sustain. Energy Rev. **152**, 111675 (2021).

[11] B. Hoen , Attitudes of US wind turbine neighbors: Analysis of a nationwide survey. Energy Policy **134**, 110981 (2019).

[12] S. Renewables, “Yougov/renewables UK survey results” (Scottish Renewables) (Tech. Rep., 2012).

[13] M. D. Heintzelman, C. M. Tuttle, Values in the wind: A hedonic analysis of wind power facilities. Land Econ. **88**, 571–588 (2012).

[14] B. Hoen , Spatial hedonic analysis of the effects of us wind energy facilities on surrounding property values. J. Real Estate Finance Econ. **51**, 22–51 (2015).

[15] A. Carr-Harris, C. Lang, Sustainability and tourism: The effect of the united states’ first offshore wind farm on the vacation rental market. Res. Energy Econ. **57**, 51–67 (2019).

[16] J. Ki , Local residents’ attitudes about wind farms and associated noise annoyance in South Korea. Energy Policy **163**, 112847 (2022).

[17] G. Pepermans, S. Rousseau, Consumers and citizens: Identity salience in choice settings focusing on local wind turbines. J. Environ. Manag. **281**, 111857 (2021). 10.1016/j.jenvman.2020.111857 33450721

[18] S. Jarvis, “The economic costs of nimbyism: Evidence from renewable energy projects” (Tech. Rep., 2021).

[19] S. Rosen, Hedonic prices and implicit markets: Product differentiation in pure competition. J. Polit. Econ. **82**, 34–55 (1974).

[20] N. V. Kuminoff, V. K. Smith, C. Timmins, The new economics of equilibrium sorting and policy evaluation using housing markets. J. Econ. Literat. **51**, 1007–1062 (2013).

[21] S. Gibbons, Gone with the wind: Valuing the visual impacts of wind turbines through house prices. J. Environ. Econ. Manag. **72**, 177–196 (2015).

[22] B. Hoen, R. Wiser, P. Cappers, M. Thayer, G. Sethi, Wind energy facilities and residential properties: The effect of proximity and view on sales prices. J. Real Estate Res. **33**, 279–316 (2011).

[23] R. J. Vyn, R. M. McCullough, The effects of wind turbines on property values in Ontario: Does public perception match empirical evidence? Canad. J. Agricult. Econ./Rev. Cana. d’agroecon. **62**, 365–392 (2014).

[24] C. Lang, J. J. Opaluch, G. Sfinarolakis, The windy city: Property value impacts of wind turbines in an urban setting. Energy Econ. **44**, 413–421 (2014).

[25] M. I. Dröes, H. R. Koster, Renewable energy and negative externalities: The effect of wind turbines on house prices. J. Urban Econ. **96**, 121–141 (2016).

[26] C. U. Jensen , The impact of on-shore and off-shore wind turbine farms on property prices. Energy Policy **116**, 50–59 (2018).

[27] M. Quentel, “Gone with the wind: Renewable energy infrastructure, welfare, papers and redistribution” (Tech. Rep. mimeo, 2023).

[28] Y. Zhao, A. Padmanabhan, S. Wang, A parallel computing approach to viewshed analysis of large terrain data using graphics processing units. Int. J. Geogr. Inf. Sci. **27**, 363–384 (2013).

[29] S. Tabik, E. L. Zapata, L. F. Romero, Simultaneous computation of total viewshed on large high resolution grids. Int. J. Geogr. Inf. Sci. **27**, 804–814 (2013).

[30] S. Tabik, A. R. Cervilla, E. Zapata, L. F. Romero, Efficient data structure and highly scalable algorithm for total-viewshed computation. IEEE J. Selected Top. Appl. Earth Observ. Remote Sens. **8**, 304–310 (2014).

[31] M. E. Data, S. Lab, “U.S. President 1976–2020” (Tech. Rep., 2017).

[32] A. Jarvis, H. Reuter, A. Nelson, E. Guevara, Hole-filled seamless SRTM data v4 (International Centre for Tropical Agriculture (CIAT), 2008). http://srtm.csi.cgiar.org.

[33] W. Guo, L. Wenz, M. Auffhammer, Supporting Documents for “The visual effect of wind turbines on property values is small and diminishing in space and time”. Github. https://github.com/weiguochina/Windmill_Visibility_House_Price_US_2024/tree/main. Deposited 24 February 2024. 10.1073/pnas.2309372121 PMC10990128 38498707

[34] Zillow Group, Zillow Transaction and Assessment Dataset (ZTRAX). Zillow. https://www.zillow.com/ztrax. Deposited 1 September 2022.

[35] B. Hoen , United States Wind Turbine Database. U.S. Geological Survey, American Clean Power Association, and Lawrence Berkeley National Laboratory data release. 10.5066/F7TX3DN0. Deposited 10 August 2022.

[36] A. Jarvis, H. Reuter, A. Nelson, E. Guevara, Hole-filled seamless SRTM data V4. International Centre for Tropical Agriculture (CIAT). https://srtm.csi.cgiar.org. Deposited 1 January 2022.

[37] MIT Election Data and Science Lab, U.S. President 1976–2020. Harvard Dataverse. 10.7910/DVN/42MVDX. Deposited 10 August 2022.

