# Supplementary material for: The visual effect of wind turbines on property values is small and diminishing in space and time
Source: Proc Natl Acad Sci U S A. 2024 Mar 18;121(13):e2309372121. doi: 10.1073/pnas.2309372121 (PMC10990128; doi:10.1073/pnas.2309372121)
Supplement: Supplementary file 1 — Appendix 01 (PDF) [file pnas.2309372121.sapp.pdf]

Supporting Information for

The visual effect of wind turbines on property values is small and diminishing in space and time

Wei Guo, Leonie Wenz, and Maximilian Auffhammer

Maximilian Auffhammer.
E-mail: auffhammer@berkeley.edu

This PDF file includes:

Supporting text
Figs. S1 to S4
Tables S1 to S5

Supporting Information Text

Robustness: Restricting building heights

Data on the average building height are obtained from the U.S. national categorical mapping of building heights from Shuttle Radar Topography Mission (SRTM). The data are a categorical mapping of estimated mean building heights, by census block group, for the conterminous United States.

The data were derived from the NASA Shuttle Radar Topography Mission, which collected "first return" (top of canopy and buildings) radar data at 30-m resolution in February, 2000 aboard the Space Shuttle Endeavor. These data were processed to estimate building heights nationally, and then aggregated to block group boundaries. Aggregation was done by calculating a zonal sum by census block group of elevations from the SRTM urban area grid, then dividing the sum by the land area of the block group in hectares . This resulted in a sum of the elevations in meters per hectare (SEPH). This dataset was assessed in three ways: (1) by comparing it by block group to the actual buildings heights of a detailed dataset from the city of San Francisco, (2) by identifying the class for 858 random points over low-density residential areas, and (3) by qualitatively checking the dataset against known tall landmarks in major cities.

Block groups were then categorized into six groups using the statistical distribution of this average elevation, namely using multiples of standard deviation from the mean as breakpoints. The categories were named "Low", "Low-Medium", "Medium", "Medium-High", "High", and "Very High". The classifications were assigned as follows: the "Low" category was assigned values where SEPH was in the range 0 to 0.5851; "Low-Medium" category: SEPH = 0.5851 to 6.9151; "Medium" category: SEPH = 6.9151 to 19.5776; "Medium-High" category: SEPH = 19.5776 to 32.24; "High" category: SEPH = 32.24 to 57.5651; and "Very High" category: SEPH greater than 57.5651. Of the 216,291 block groups, 33.5% are "Low", 36.7% are "Low-Medium", 20.0% are "Medium", 6.5% are "Medium-High", 2.5% are "High", and 0.8% (1,722) are "Very High". Block groups categorized as "Very High" tend to be focused in a small number of the very densest cities, such as Manhattan and Los Angeles. From these means and standard deviations we also roughly make an estimate of how tall and how many stories buildings typically would have in each category. Exact number of meters per story varies widely, so an estimate of 3.5 was used, based on a height per story of 10-12 feet. To demonstrate the robustness of our overall results, we focus on areas with low and low to medium height buildings where the Low category of building heights is defined as primarily 1-2 story buildings and the Low-Medium category as primarily 2-3 story buildings. Figure S1 presents the spatial distribution of the average building height categories. The majority of places fall in the group of "Low‘' and "Low-Medium" groups. The results are almost identical to our main specification in Table (1). The full regression results are given in Table (S2).

Data Acquisition and Replication

To ensure reproducibility of our results, below we provide a comprehensive guide to acquiring and using the data essential for replicating our study.

A. Data Acquisition.

ZTRAX data. ZTRAX data is offered by Zillow's research team through their website https://www.zillow.com/research/ztrax/. To access ZTRAX data, the user will need to first review and agree to the ZTRAX Data License Agreement, then complete the registration online. Once logged in, the user can request access to specific ZTRAX datasets. For acquisition, the user will need to be prepared to provide details on the intended use of the data. To replicate of our study, we recommend applying for comprehensive access to ZTRAX's housing transaction data, covering all U.S. states from 1995 to the most recent available data.

Windmill data. Windmill data can be accessed from the United States Wind Turbine Database (USWTDB) produced by USGS, available at https://eerscmap.usgs.gov/uswtdb/data/. We have consolidated key data and saved it as "windmill.dta" in the replicate kit.

Digital Elevation Models. Digital Elevation Models are produced by NASA's Shuttle Radar Topographic Mission and available at https://srtm.csi.cgiar.org/. These are crucial for viewshed computations in the replication. Due to size constraints, the original DEMs are not included as part of our replication kit. The user will need to download and extract the data to "/data/DEMs/" under the replication directory.

Other Data. Data for heterogeneity analysis are drawn from multiple sources. The county-level median household income records come from the 2015 American Community Survey. Presidential election data is sourced from the MIT Election Data and Science Lab. Additionally, the county-level average elevation data is derived from the Environmental Systems Research Institute. All these data are either acquired through public access in our code or included in the replication kit.

B. Software and Libraries. The analysis in this paper are processed on a server with 16 cores, 32 threads, 3.10GHz CPU, and 256 GB of memory. The following software and related libraries are required:

1. R version 4.2.1, with the following libraries and their versions:
   leafem 0.2.0, colorspace 2.0-3, deldir 1.0-6, class 7.3-20, leaflet 2.2.0, satellite 1.0.4, base64enc 0.1-3, fs 1.5.2, rstudioapi 0.14, proxy 0.4-27, roxygen2 7.2.1, listenv 0.8.0, hexbin 1.28.2, remotes 2.4.2, fansi 1.0.3, xml2 1.3.3, codetools 0.2-18,

doParallel 1.0.17, cachem 1.0.6, knitr 1.40, pkgload 1.3.0, jsonlite 1.8.2, png 0.1-7, shiny 1.7.2, BiocManager 1.30.22 compiler 4.2.1, assertthat 0.2.1, fastmap 1.1.0, cli 3.6.1,, later 1.3.0, htmltools 0.5.3, prettyunits 1.1.1 tools 4.2.1, glue 1.6.2, vctrs 0.6.1, iterators 1.0.14, crosstalk 1.2.0, lwgeom 0.2-8, xfun 0.33,, stringr 1.4.1, globals 0.16.1, ps 1.7.1, mime 0.12, miniUI 0.1.1.1, lifecycle 1.0.3, devtools 2.4.5, terra 1.5-34, zoo 1.8-11, hms 1.1.3, promises 1.2.0.1, parallel 4.2.1, RColorBrewer 1.1-3 yaml 2.3.5, memoise 2.0.1, latticeExtra 0.6-30 stringi 1.7.8, e1071 1.7-11, pkgbuild 1.3.1, rlang 1.1.0, pkgconfig 2.0.3, evaluate 0.17, purrr 0.3.5, htmlwidgets 1.5.4 processx 3.7.0, tidyselect 1.2.0 parallelly 1.32.1 magrittr 2.0.3, R6 2.5.1, generics 0.1.3, profvis 0.3.7, DBI 1.1.3, pillar 1.8.1, units 0.8-0, tibble 3.1.8, crayon 1.5.2, interp 1.1-3, KernSmooth 2.23-20 utf8 1.2.2, rmarkdown 2.17, urlchecker 1.0.1 jpeg 0.1-10, progress 1.2.2, usethis 2.1.6, grid 4.2.1, callr 3.7.2, digest 0.6.31, classInt 0.4-7, webshot 0.5.4, xtable 1.8-4, httpuv 1.6.6, stats4 4.2.1, munsell 0.5.0, viridisLite 0.4.1, sessioninfo 1.2.2
2. ArcGis version 10.7.2, and ArcGis business analyst, with the USA Local Composite locator included.
3. Grass GIS version 7.8.3.

C. Data Pre-processing.

Geocoding of ZTRAX dataset. As ZTRAX data may contain coordinates that do not accurately represent a property's true location, we conduct a comprehensive geocoding process to obtain a geospatial dataset with accuracy.

The geocoding process translates a street address into its precise latitude and longitude coordinate. It is executed through ArcGIS 10.7.2 and the Business Analyst toolbox. A detailed guide on how to perform geocoding is provided by the ArcGIS website at https://desktop.arcgis.com/en/arcmap/latest/manage-data/geocoding/geocoding-a-table-of-addresses-in-arcmap.htm. The user will need to load the "USA Local Composite locator" when asked to select geolocator. In our tests, the geocoding program processed nearly 1 million addresses within 2 hours.

Viewshed Calculations. The viewshed calculations for all windmills are processed through an integration between GRASS GIS with R software. In the replication kit, we provide a replication code file ("/program/1-calculate_viewshed.R") that completes these calculates. A brief outline of the calculation process is as follows:

1. Import the windmill data and the DEMS into GRASS GIS. The DEMs are segmented into 30 degree by 30 degree tiles in their original format.
2. Re-project the DEMs tiles to ESPG:3857 (WGS Pseudo Mercator in meter unit), and merge the tiles into a singular surface raster map.
3. Convert the windmill data into spatial format and re-project it to the same projection.
4. Partition the windmill data into 100km by 100km grids for parallel purpose. This results in 480 grids each of which containing at least one wind turbine.
5. Iterate the viewshed calculation over the grids sequentially. For each grid, define a buffer within 100km radius of the grid and set it as the analysis region. Then compute the viewsheds for wind turbines inside the grid in parallel, using the windmill's hub height, setting the observer's height as 1.75m, designating the maximum visibility distance as 10km, and factoring in the Earth's curvature.
6. Export the raster map of viewsheds, which identifies areas from where a wind turbine is visible. Save the viewshed raster in output.

In our tests, the viewshed calculation of a sample wind turbine took a few seconds. We established an integration of GRASS GIS with R to automate this calculation process and facilitate parallel programming. The cumulative size of viewshed raster files is 91.9 gigabytes for all windmills in the sample.

Mapping Property to Viewsheds. We map properties with windmill viewsheds based on their geocoded location. Encompassing more than 300 million housing transactions and viewshed rasters for over 60,000 wind turbines, this poses substantial computational challenge. To manage this, we employ parallel processing for the mapping operation. We identify 6 key variables that will later determine the DiD indicators. These include: 1) the visibility of a wind turbine from the property at the time of the transaction; 2) the property's potential visibility of a wind turbine if transacted today; 3) the distance to the nearest visible wind turbine at the time of the transaction; 4) the distance to the nearest wind turbine, regardless of visibility, at the time of the transaction; 5) the distance to the nearest visible wind turbine as of today; and 6) the distance to the nearest wind turbine, regardless of visibility, as of today. In the replication kit, we provide a file of replication code ("/program/2-map_ztrax.R") that completes the mapping process.

D. Data Analysis. We provided the replication codes ("/program/3_1-turbine_data_summ.R", "/program/3_2-ztrax_data_summ.R", and "/program/4_reg.R") that generate all the figures and tables in the main text and the SI. Note that to compile the file, the user will need to acquire a token from the census website and add it to the program.

Supporting Figures

Fig. S1. Average Building Height by Census Block

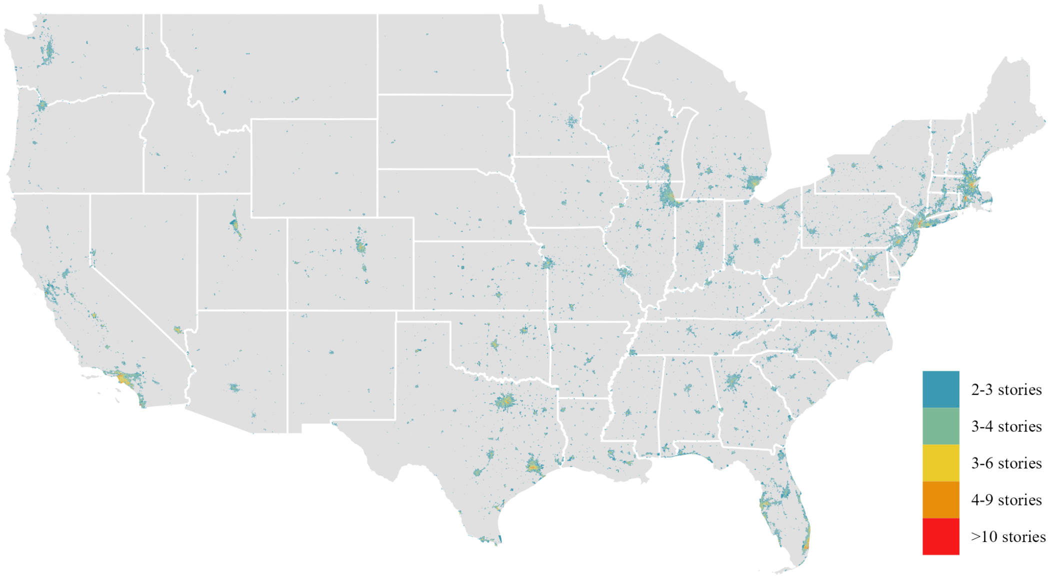

Source: U.S. national categorical mapping of building heights from Shuttle Radar Topography Mission

Fig. S2. Number of Wind Turbines In View

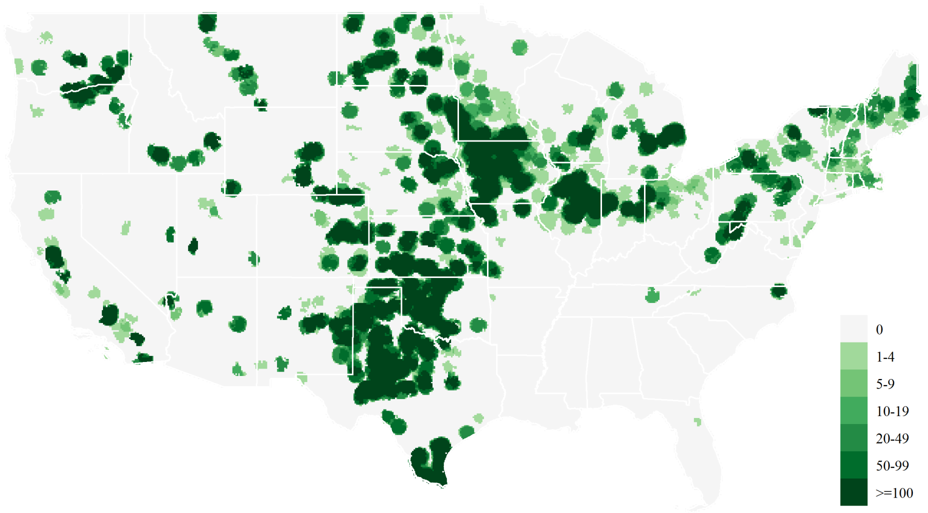

Source: estimation based on the wind turbines site, the wind turbines height, and the digital elevation models of the nation.

Fig. S3. Test on Parallel Pre-Treatment Trend

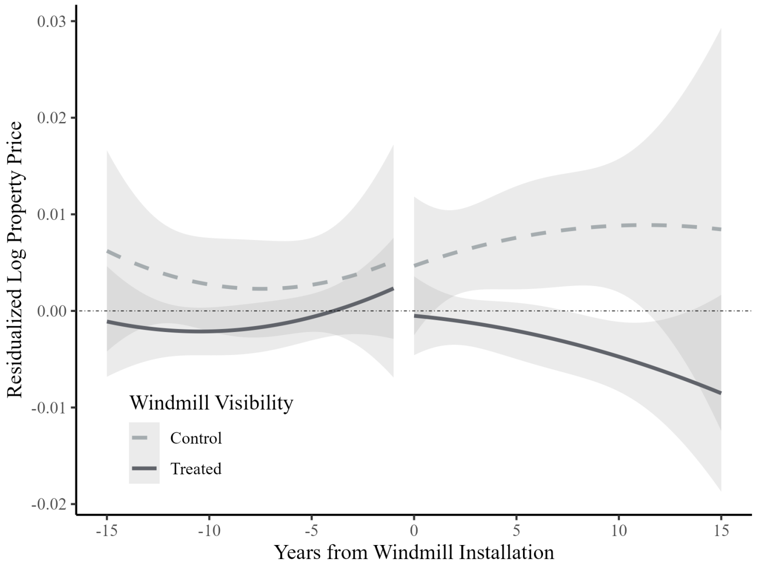

Note: The residualized property value variable on RHS is obtained by regressing the log of property value on property characteristics and the fixed effects controls in the baseline regression and using the residualized component. The fitted curves are computed using quadratic smoothing functions on the residualized property value variable as a function of the number of years from windmill installation, for the control and treated groups respectively.

Fig. S4. Share of Treated and Treated-Post-Treatment Properties by State

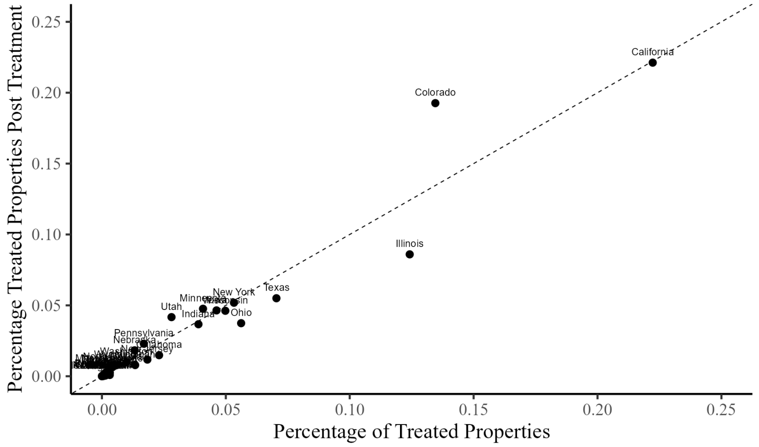

Note: Each scatter point represents a state. The x-axis displays the share of treated properties by state, defined as the proportion of property transactions within visible areas of wind turbines relative to the total number of transactions in the visible areas. The y-axis shows the post-treatment share of treated properties by state, determined by the number of property transactions within wind turbine visible areas after installation as a share of the total transactions in these areas post-installation.

Supporting Tables

Table S1.
Robustness Tests

	Baseline (1)	Disclosure (2)	Repeated Sales (3)	Prop × Year (4)	Prop × State (5)
Post × Treated	-0.0115***	-0.0137***	-0.0281***	-0.0129***	-0.0107**
	(0.00428)	(0.00453)	(0.00721)	(0.00434)	(0.00411)
Post-Treatment	0.00879	0.0105*	0.0717**	0.00988	0.00917
	(0.00586)	(0.00603)	(0.0312)	(0.00703)	(0.0057)
Treated	-0.0101**	-0.00906*	0.0692	-0.00965**	-0.0098**
	(0.0044)	(0.00484)	(0.0696)	(0.00458)	(0.00432)
Year Built	0.00302***	0.00299***			
	(0.000205)	(0.000204)			
# Bedrooms	0.0381***	0.0378***			
	(0.00204)	(0.00212)			
# Bathrooms	0.148***	0.149***			
	(0.00457)	(0.00501)			
Lot Size Acres	8.92e-06	8.49e-06			
	(7.67e-06)	(7.51e-06)			
N	5552911	5139261	1652537	5492914	5552911
Adj. R^2	0.512	0.516	0.455	0.516	0.513
FE: Tract-by-Year	X	X	X	X	X
FE: County-by-Month	X	X	X	X	X
FE: Parcel ID			X		
FE: Prop Char × Year				X	
FE: Prop Char × State					X
Std. Errors Clustered at Census Tract and Year Level					

Note: Estimation results for the property value on the effect of wind turbine visibility, using different specifications and samples. Dependent variable is the log of sales price. Column (1) the baseline specification. Column (2) excludes non-disclosure states of ZTRAX database, including Alaska, Idaho, Indiana, Kansas, Louisiana, Mississippi, Montana, New Mexico, North Dakota, South Dakota, Texas, Utah, and Wyoming (Wentland et al. 2020). Column (3) limits to properties with repeated sales only and controls for the property fixed effects accordingly. Column (4) and (5) expands the baseline specification by adding the controls for property characteristics interacted with the sales year indicators and with the state indicators, respectively. Each specification controls for a full set of property characteristics and fixed effects of the census tract by sales year level and the county by sales month level. Standard errors are clustered twoway at the census tract and year level. * p<0.1, ** p<0.05, *** p<0.01.

Table S2.
Single, Double, Triple and Quadruple Differences Specifications

	Single Diff		DiD	Tri. Diff	Quad. Diff
	(1) 10km	(2) 50km	(3) 10km	(4) 50km	(5) 50km
Post-Treatment (Visibility)			0.00896	2.84e-05	0.00313
			(0.00589)	(0.00568)	(0.00646)
Treated (Visibility)	-0.0162***	-0.0162***	-0.0101**	-0.0124***	-0.0115***
	(0.00397)	(0.00394)	(0.00439)	(0.00411)	(0.00424)
Proximate		-0.0116		-0.0132	-0.017*
		(0.00761)		(0.00807)	(0.00868)
Post-Proximity					0.0016
					(0.0172)
Proximate × Post-Proximity					0.0107
					(0.0111)
× Post-Treatment				0.00207	-0.00353
				(0.00574)	(0.00759)
× Treated × Post-Treatment			-0.0112**	-0.00827*	-0.00899**
			(0.00431)	(0.00428)	(0.00429)
N	5705597	30398120	5705597	30398120	30398120
Adj. R^2	0.516	0.523	0.516	0.523	0.523
FE: Census Tract × Sales Year	X	X	X	X	X
FE: County × Sales Month	X	X	X	X	X
Std. Errors Clustered at Census Tract and Year Level					

Note: Estimation results for the property value on the effect of wind turbine visibility, using different specifications. Dependent variable is the log of sales price. Column (1) uses all transactions within 10 km from wind farms and applies a single difference framework that compares between visible and non visible areas. Column (2) uses all transactions within 50 km from wind farms and incorporates another cross-sectional difference by proximity (within 10 km). Column (3) applies our baseline specification of spatial DiD model on all transactions within 10 km from windmills. Column (4) expands the sample size to all transactions within 50 km and adds an interaction term with the indicator for proximity. Column (5) further incorporates another interaction dimension with the indicator for the transaction after the wind turbine installation in proximity. Each specification controls for a full set of property characteristics and fixed effects of the census tract by sales year level and the county by sales month level. Standard errors are clustered twoway at the census tract and year level. * p<0.1, ** p<0.05, *** p<0.01.

Table S3.
Robustness Test: Average Building Height

**Low Building Blocks**	Low (≤ 1 Stories)		Low-Medium (1-2 Stories)	
	All	Urban	All	Urban
	(1)	(2)	(3)	(4)
Treated × Post-Treatment	-0.0141**	-0.0118*	-0.015**	-0.0159**
	(0.00583)	(0.00656)	(0.00732)	(0.00691)
Post-Treatment	0.00676	0.00305	0.0252***	0.0254***
	(0.00861)	(0.00856)	(0.00951)	(0.00937)
Treated	-0.0151**	-0.0125*	0.00864	0.0101
	(0.00634)	(0.00698)	(0.00803)	(0.00794)
N	2417916	2021891	1515155	1496666
Adj. R^2	0.467	0.476	0.536	0.537
FE: Census Tract × Sales Year	X	X	X	X
FE: County × Sales Month	X	X	X	X
Std. Errors Clustered at Census Tract and Year Level				

Note: Robustness tests for the property value on the effect of wind turbine visibility, by average building height within the census block. Block groups are categorized into four groups using the statistical distribution of the sum-elevations per hectare and comparing it with the average height of buildings by the number of stories. Each specification controls for a full set of property characteristics and fixed effects of the census tract by sales year level and the county by sales month level. Standard errors are clustered twoway at the census tract and year level. * p<0.1, ** p<0.05, *** p<0.01.

Table S4.
Summary Statistics of Wind Turbines.

	N	Mean	SD	Max	Min
Operational Year	68,649	2011.15	8.01	2021	1981
# Turbines on site	68,649	105.41	95.41	731	1
Cumulative Capacity (mw)	68,649	167.41	103.3	525.02	0.05
Turbine Capacity (kw)	68,649	1926.08	711.38	6000	50
Hub Height (m)	68,649	80.26	12.47	131	22.8
Rotor Diameter (m)	68,649	94.41	23.86	155	13.4
Rotor Swept Area (m^2)	68,649	7447.13	3289.6	18869.2	141.03
Total Height (m)	68,649	127.63	22.95	199.6	30.4
Retrofit = 1	68,649	0.09	0.28	1	0

Note: Summary Statistics of Wind Turbine Installations. The variables reported are: the year of which the wind project (plant) became operational, the number of wind turbines on site within the plant, the cumulative capacity of all turbines on site, the wind turbine's capacity, the turbine height from the hub, the turbine rotor diameter, the turbine rotor swept area, the turbine total height, and the indicator for whether the turbine has been retrofitted.
The top five states for wind turbine installation are: Texas (24.7%), California (9.5%), Indiana (8.7%), Oklahoma (6.8%), and Kansas (5.4%). The majority of wind turbines are located in rural areas (99.8%).
Source: United States Wind Turbine Database (2022 Version) of USGS.

Table S5.
Summary Statistics of Housing Transactions.

	All (N=54,415,117)		Visible (N=9,282,588)		Non-Visible (N=45,132,529)		Mountainous (N=16,079,661)		Non-Mountainous (N=38,335,456)	
	Mean	SD	Mean	SD	Mean	SD	Mean	SD	Mean	SD
Sales Year	2008.2	6.61	2012.11	5.43	2007.4	6.54	2007.44	6.56	2008.58	6.62
Sales Price ($)	234484	256268	235914	249986	234190	257540	305922	279620	206261	243234
Lot Size (sq ft)	37971	173684	28148	140675	40122	180035	21607	107878	46278	199590
Year Built	1972.57	30.79	1973.57	31.6	1972.36	30.61	1978.31	25.06	1973.65	32.40
# Rooms	4.73	3.68	4.96	3.77	4.68	3.65	5.61	3.60	4.23	3.63
# Bedrooms	2.92	1.33	3	1.27	2.91	1.34	3.26	1.06	2.74	1.43
# Bathrooms	1.85	1.04	1.86	1.03	1.85	1.05	2.15	0.84	1.69	1.11
# Vis. Turbines	8.61	56.14	50.45	127.93	0	0	17.89	92.37	4.79	29.65
Dist. Vis. Turbine (m)	16623	9703	16623	9703			17806	10283	16052	9359
# Turbines <50km	66.27	215.68	172.89	371.83	44.34	157.57	152.07	353.97	30.84	99.02
Dist. Turbine (m)	24725	12614	15276	8835	28648	11845	26656	13041	23717	12291

Note: Summary statistics of housing transactions for the period of 1997-2020, within 50 km from wind turbine sites. The variables reported are: the year of transaction, the sales price, the lot size on which the property is located, the effective year built of the property, the number of rooms, the number of bedrooms, the number of bathrooms, the number of visible wind turbines on the time of transaction, the distance to the nearest visible wind turbine on the time of transaction, the number of wind turbines within 50km, the distance to the nearest wind turbines on the time of transaction. The sample is segmented by whether it can view at least one wind turbine on the time of transaction.
Among all housing transactions within 10km from wind turbine sites, 17.1% can view a wind turbine at the time of transaction.
The top 5 states for housing transactions affected by windmill visibility are: California (22.2%), Colorado (19.2%), Illinois (8.7%), Texas (5.1%), and New York (5.1%).
The top 5 states for housing transactions non-visible to windmill are: California (18.8%), Illinois (15.9%), Texas (11.2%), New York (5.9%), and Wisconsin (5.5%).
Source: Zillow's ZTRAX database (2021 version).
